# Effect of Valproic Acid on Promoting the Differentiation of Human Embryonic Stem Cells Into Cholangiocyte-Like Cells

**DOI:** 10.1093/stcltm/szad079

**Published:** 2023-11-23

**Authors:** Shuai Deng, Xiaoyu Zhao, Ziyan Kou, Yanlun Zhu, Xuerao Zhang, Hon Fai Chan

**Affiliations:** Laboratory of Molecular Pharmacology, Department of Pharmacology, School of Pharmacy, Southwest Medical University, Luzhou, People’s Republic of China; Key Laboratory for Regenerative Medicine of the Ministry of Education of China, School of Biomedical Sciences, Faculty of Medicine, The Chinese University of Hong Kong, Shatin, Hong Kong SAR, People’s Republic of China; Institute for Tissue Engineering and Regenerative Medicine, The Chinese University of Hong Kong, Shatin, Hong Kong SAR, People’s Republic of China; Key Laboratory for Regenerative Medicine of the Ministry of Education of China, School of Biomedical Sciences, Faculty of Medicine, The Chinese University of Hong Kong, Shatin, Hong Kong SAR, People’s Republic of China; Institute for Tissue Engineering and Regenerative Medicine, The Chinese University of Hong Kong, Shatin, Hong Kong SAR, People’s Republic of China; Key Laboratory for Regenerative Medicine of the Ministry of Education of China, School of Biomedical Sciences, Faculty of Medicine, The Chinese University of Hong Kong, Shatin, Hong Kong SAR, People’s Republic of China; Institute for Tissue Engineering and Regenerative Medicine, The Chinese University of Hong Kong, Shatin, Hong Kong SAR, People’s Republic of China; Key Laboratory for Regenerative Medicine of the Ministry of Education of China, School of Biomedical Sciences, Faculty of Medicine, The Chinese University of Hong Kong, Shatin, Hong Kong SAR, People’s Republic of China; Institute for Tissue Engineering and Regenerative Medicine, The Chinese University of Hong Kong, Shatin, Hong Kong SAR, People’s Republic of China; Center for Neuromusculoskeletal Restorative Medicine, Hong Kong SAR, People’s Republic of China; Key Laboratory for Regenerative Medicine of the Ministry of Education of China, School of Biomedical Sciences, Faculty of Medicine, The Chinese University of Hong Kong, Shatin, Hong Kong SAR, People’s Republic of China; Institute for Tissue Engineering and Regenerative Medicine, The Chinese University of Hong Kong, Shatin, Hong Kong SAR, People’s Republic of China; Key Laboratory for Regenerative Medicine of the Ministry of Education of China, School of Biomedical Sciences, Faculty of Medicine, The Chinese University of Hong Kong, Shatin, Hong Kong SAR, People’s Republic of China; Institute for Tissue Engineering and Regenerative Medicine, The Chinese University of Hong Kong, Shatin, Hong Kong SAR, People’s Republic of China; Center for Neuromusculoskeletal Restorative Medicine, Hong Kong SAR, People’s Republic of China; Hong Kong Branch of CAS Center for Excellence in Animal Evolution and Genetics, Hong Kong SAR, People’s Republic of China

**Keywords:** valproic acid, hESC, differentiation of cholangiocyte, Notch signaling pathway

## Abstract

Cholangiocytes form a complex 3D network of bile ducts in the liver and contribute to liver function. The damage or destruction of cholangiocytes can lead to biliary diseases, and the shortage of cholangiocytes remains an obstacle for drug development targeting biliary diseases. Valproic acid (VPA) is a potent activator of Notch signaling pathway that is essential for cholangiocyte differentiation. Here, we report a VPA-based approach for cholangiocyte differentiation of human pluripotent stem cells. VPA activated Notch2 expression and upregulated *HES-1*, *HEY-1*, and *Sox9* gene expression in hESC-derived hepatoblast. After 7 days treatment, VPA promoted successful differentiation of hepatoblast into cholangiocytes expressing cholangiocyte marker genes (*AE2*, *AQP1*, *CFTR*) and proteins (CK19 and CK7). In addition, the differentiated cholangiocytes formed bile duct-like structures after implantation into the spleen of NOD/SCID mice. Our results suggested that VPA can promote hESC differentiation to cholangiocyte-like cells. The induced cholangiocytes may serve as a potential cell source for both in vitro modeling and regenerative therapy of cholangiopathies. The findings can also support further development of small-molecule based differentiation protocols for cholangiocyte production.

## Graphical Abstract

**Graphical Abstract:**
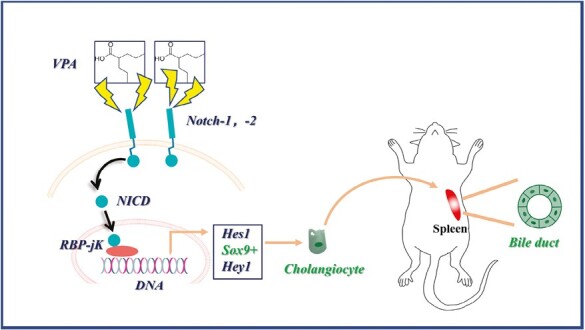

**Significance Statement**

In this work, we reported the effect of valproic acid (VPA) treatment on promoting hepatoblast differentiation into cholangiocyte-like cells for the first time. Our studies contribute to the generation of cholangiocyte-like cells via a small-molecule-based approach which can facilitate the studying and development of therapies for biliary diseases.

## Background

Cholangiocytes, also called biliary epithelial cells, form a complex 3D network of bile ducts in the liver.^1^ In humans, the total length of biliary network reaches approximately 2 km.^2^ It is responsible for the collection and transportation of bile acids secreted by hepatocytes.^3^ In addition, cholangiocytes also contribute to liver function. For example, cholangiocytes are involved in the hormone-regulated bile secretion and mucin secretion.^4^ Cholangiocytes also participate in liver regeneration after liver injury.^5^ Unfortunately, the damage or destruction of cholangiocytes can lead to biliary diseases which are difficult to treat and can lead to end-stage liver disease that are associated with high mortality.^6^

A variety of factors (such as chronic cholestasis, infection, and toxic injury) can increase the risk of development of biliary diseases.^6,7^ However, the pathogenesis of biliary diseases is still unclear due to the shortage of in vitro model.^8^ In addition, the shortage of in vitro biliary model also limits the progress of drug development targeting biliary diseases. Therefore, there is an urgency to generate sufficient cholangiocytes for the construction of in vitro biliary model that will facilitate the study of biliary diseases and screening of drug candidates. So far, many attempts have been investigated to culture primary cholangiocyte in vitro with little success. This is because cholangiocytes only account for approximately 3% of total liver cells, and the isolation of pure cholangiocytes from liver biopsies is challenging.^9,10^ Recently, using human pluripotent stem cells, including human-induced pluripotent stem cells (hiPSC) and human embryonic stem cells (hESC), to generate cholangiocyte-like cells has been investigated.

Cholangiocyte and hepatocyte share common precursors called hepatoblast. Hepatoblasts exhibit bipotent characteristics and can differentiate into hepatocytes or cholangiocytes.^11,12^ A 3-step differentiation protocol mimicking liver development, including definitive endoderm differentiation, hepatic specification, and hepatocyte maturation, has been widely used to generate hepatocytes via an intermediate step of hepatoblast production.^13,14^ In contrast, only few studies have attempted to generate cholangiocyte from hepatoblast derived from hESC and hiPSC.^15,16^ Previous studies have reported that activation of Notch pathway can initiate hepatoblast differentiation into cholangiocyte, as Notch signaling is required to establish cholangiocellular fate during liver development.^17^ Notch pathway activation induces the expression of the downstream Hes/Hey gene family, as well as the SOX9 gene, where SOX9 is a specific and early-stage marker of biliary cells in developing liver and regulates bile duct morphogenesis.^18,19^ In addition, Hes/Hey gene family also play important roles in regulating hepatoblast biliary differentiation. Specifically, Hes1/Hey1 act as transcriptional repressors by binding to specific DNA sequences and inhibiting the expression of genes involved in hepatocyte differentiation while promoting the differentiation of biliary cells.^19-21^ To promote biliary lineage commitment from hepatic progenitor cells, a number of growth factors and cytokines (such as EGF, HGF, TGFβ, and IL6) have been reported.^18,22,23^ However, the high cost and short half-life of these biological factors would limit the mass production of cholangiocyte. In contrast, a small-molecule based approach can be advantageous in generating cholangiocytes.

Valproic acid (VPA, 2-propylpentanoic acid) is an established small-molecule drug in epilepsy therapy with minimal cytotoxicity.^24^ Recently, it has also been reported that VPA can activate different signaling pathways in addition to treating epilepsy. For example, VPA can promote hair regrowth by activating the Wnt/β-catenin pathway.^25,26^ In addition, previous studies have reported that VPA can activate Notch signaling cascade by increasing the levels of intracellular Notch and Hes-1, and the role of VPA in promoting neural differentiation by activating the Notch signaling cascade has been extensively reported.^27,28^ For example, Vukićević *et al* reported the treatment of VPA enhances the neuronal differentiation of sympathoadrenal progenitor cells by upregulating Notch2 and Hes-1[27]. Given that Notch2 signaling pathway also plays an important role in cholangiocyte differentiation, whether VPA can promote the differentiation of hepatic progenitor cells into cholangiocyte remains to be studied. Based on the reported effect of VPA on the regulation of Notch2 signaling pathway, we hypothesized that VPA can initiate the cholangiocyte differentiation of hepatoblasts.

In this study, we report a VPA-based approach for cholangiocyte differentiation of human pluripotent stem cells. hESC were differentiated to hepatoblast by a standard differentiation protocol followed by the treatment with VPA. VPA activated the Notch2 expression and upregulated *HES-1*, *HEY-1*, and *SOX9* gene expression. After 7 days treatment, the hepatoblasts differentiated into cholangiocytes and expressed cholangiocyte marker genes (*AE2*, *AQP1*, *CFTR*, etc.) and proteins (CK19 and CK7). The bile duct-like structures were observed after the cholangiocytes were implanted into the spleen of NOD/SCID mice. Our results suggested that VPA-induced cholangiocyte may serve as a potential cell source for both in vitro modeling and regenerative therapy of cholangiopathies. Overall, we demonstrated that VPA could induce the differentiation of hepatoblast to cholangiocyte. The findings support further development of small-molecule based differentiation protocols for cholangiocyte production.

## Methods

### hESC culture and generation of hepatoblast

Culture and expansion of hESC: The hESC line cell, H9 at passages 5-10, were regularly tested mycoplasma free using mycoplasma test kit (Vazyme, China), and were maintained under feeder-free culture condition on growth factor reduced Matrigel (0.125 mg/mL) (Corning, USA) in mTeSR1 medium (STEMCELL Technologies, Canada). For hESC passaging and expansion, when hESC reached a confluence level of ~80%, hESC colonies were dissociated with Accutase (Life Technologies, USA) and resuspended as single cells in mTeSR1 medium containing 10 μM Y-27632 (Millipore, USA) and seeded in wells coated with growth factor reduced Matrigel. hESC were routinely passaged every 3-4 days.

Generation of hepatoblast from hESC: The generation of hepatoblast from hESC involves definitive endoderm differentiation and hepatic specification. For definitive endoderm differentiation, differentiation began around 18 to 20 hours after hESC seeding on Matrigel-coated surface. hESC were incubated with DE Induction Medium A (Thermofisher, USA) for the first 24 hours and then DE Incubation Medium B (Thermofisher) for another 24 hours to induce definitive endoderm differentiation. Then the cells were cultured for 5 days for hepatoblast specification in Knockout DMEM basal medium (Thermofisher) containing 20% KOSR (Thermofisher) supplemented with 1% NEAA (Thermofisher), 0.5% GlutaMAX (Thermofisher), 0.2% β-mercaptoethanol (Thermofisher), 1% Penicillin-Streptomycin (Thermofisher), and 1% Dimethylsulfoxide (Thermofisher), and the medium was replaced every 48 hours.

### Cholangiocyte-Like Cell and Hepatocyte Differentiation From Hepatoblast Cell

Following endoderm differentiation and hepatoblast specification, the generated hepatoblasts were cultured in VPA free (VPA−) or VPA addition (VPA+) medium for further hepatocyte or cholangiocyte differentiation for 10 and 7 days, respectively. Hepatic differentiation medium was based on HepatoZYME medium (Thermofisher) containing 5% KOSR, 1% hydrocortisone 21-hemisuccinate (Sigma, USA), 1% GlutaMAX, 1% Pis, 1% dimethylsulfoxide, 10 ng/mL hepatocyte growth factor (Peprotech, USA), and 20 ng/mL oncostatin M (Peprotech). A 2-mM VPA concentration was used for biliary differentiation, which was based on previous reports which demonstrated effective activation of Notch signaling pathways.^27^ The medium was replaced every 72 hours.

### RNA Extraction and Real-Time Quantitative PCR

Total RNAs were extracted with TRIzol reagent (Invitrogen, USA). cDNA was synthesized from 1000 ng of total RNA template using HiScript III RT SuperMix with gDNA Eraser (Vazyme). Real-time quantitative PCR was performed with TB Green Premix Ex Taq II (TAKARA, Japan) using the Quant Studio 7 (QS7, USA) Flex Real Time PCR System (Applied Biosystems, USA). Primers used are listed in Supplementary Table S1. Results were normalized to that of GAPDH and quantification of relative expression was determined by the 2^−ΔΔCt^ method.

### Immunofluorescence Staining

Cells were fixed with 4% paraformaldehyde (PFA, Sigma) for 15 minutes at room temperature, before permeabilized with 0.3% Triton X-100 (Sigma) for 5 minutes and blocked with 5% bovine serum albumin (BSA) for 30 minutes for immunostaining. Then the cells were incubated with primary antibodies in 1% BSA at 4 ℃ overnight, and secondary antibodies in 1% BSA for 1 hour at room temperature. Nuclear staining was performed with DAPI (Sigma) via incubation for 15 minutes. Finally, the samples were washed 3 times with PBS for 10 minutes at room temperature. All immunofluorescence images were acquired using a Leica TCS-SP8 confocal microscope. LAS X software (Leica) was used for image processing. A complete list of the primary and secondary antibodies used is provided in Supplementary Table S2.

### Rhodamine 123 Uptake Assay

Cells were incubated with 100 μM rhodamine 123 (Sigma-Aldrich) in HepatoZYME medium for 30 minutes at 37 ℃ and washed 3 times with cell culture medium. Then, the cells were incubated at 37 ℃ for another 40 minutes. Images were acquired using a Nikon Inverted Microscope (ECLIPSE Ti2-A).

### Intrasplenic Implantation

Non-obese diabetic severe combined immunodeficient (NOD-SCID) mice of 6-8 weeks were purchased from the Laboratory Animal Services Centre (CUHK, Hong Kong). All animal experiments were performed with the approval of the Animal Experimentation Ethics Committee of the Chinese University of Hong Kong (Ref. No. 21-249-MIS). The mice were anesthetized with ketamine (50 mg/mL, Bayer, Germany) and xylazine (20 mg/mL, Bayer, Germany). Then the skin of splenic area was transversely dissected and a small incision was created through the peritoneal layer to expose the spleen. The spleen was gently lifted before 10^5^ cholangiocytes were implanted into spleen with tweezer. Sham operation was performed by introducing PBS. The peritoneal cavity was then closed by suturing. Regular post-operative care was performed.

### Hematoxylin Eosin Staining and Immunohistochemistry

Spleen samples from different groups were fixed with 4% PFA at 4 ℃ overnight, washed with PBS and then embedded in Tissue Processor. The samples were dehydrated, embedded in paraffin, and sectioned at 5 μm thickness. Paraffin-embedded sections were dewaxed with xylene and rehydrated. Hematoxylin eosin (H&E) staining were performed in accordance with the manufacturer’s instructions. Antigen retrieval was performed in Antigen Retriever (PT Module, Thermofisher). The subsequent immunohistochemistry staining steps were performed as described above (Immunofluorescence staining).

### Statistical Analysis

Data were expressed as mean ± standard deviation (SD), and each experiment was repeated 3 times. Statistical analysis was performed on the results of different groups by one-way ANOVA. All the statistical analyses were carried out using the GraphPad Prism software version 8.0.1. Statistically significant values were defined as *P* < 0.05.

## Results

### Generation and Characterization of Hepatoblast

**Figure 1.**
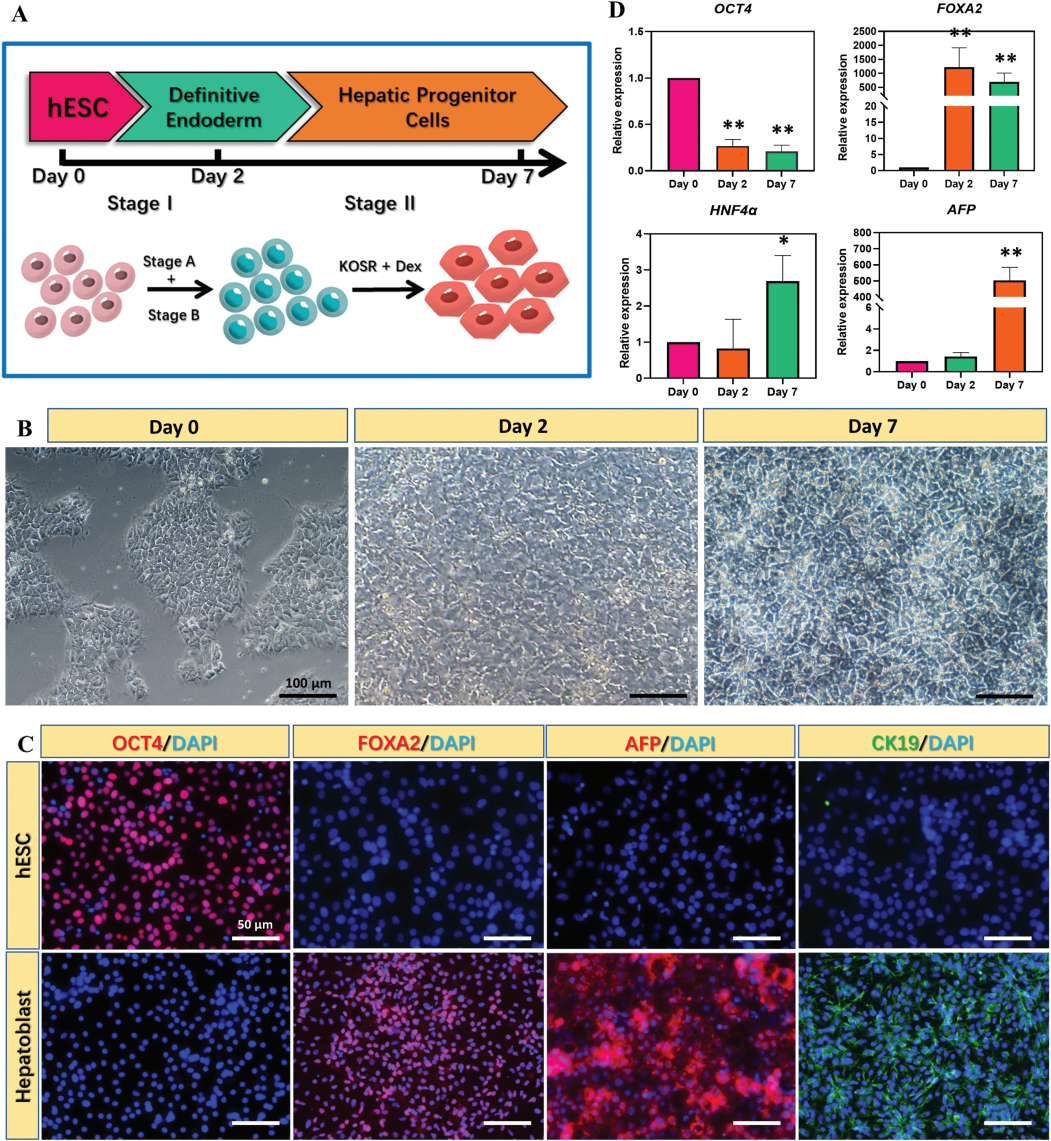
Differentiation of hESC into hepatoblasts. (**A**) Protocol of hepatic specification of hESC. (**B**) Bright field images of hESC during hepatic specification. (**C**) Immunostaining of marker proteins representative of different differentiation stages at various time points. (**D**) Expression of marker proteins representative of different differentiation stages at various time points. (*n* = 3, * indicates *P* < .05, **P < .01 vs. day 0)

Hepatoblasts are the common precursors of hepatocytes and cholangiocytes and can give rise to both cell types. In this study, hESC were differentiated to hepatoblasts using a published differentiation method as illustrated in Fig. 1A.^13^ Following hepatic specification, the morphology of hESC gradually changed from defined colonies to less dense and flatter cells containing prominent nuclei (Fig. 1B). The immunostaining results suggested that more than 80% of total cells on Day 7 expressed hepatic transcription factor HNF4α, which is crucial to regulate hepatogenesis, as well as hepatoblast markers AFP (~90% of total cells) and CK19 (~90% of total cells). On the contrary, more than 90% of cells on day 0 expressed pluripotency marker OCT4 but not HNF4α, AFP, and CK19. On day 7, OCT4 was undetectable (Fig. 1C). These results were further confirmed by qPCR analysis (Fig. 1D). The qPCR results also showed that *OCT4* gene was highly expressed on day 0 and decreased during 7 days of differentiation, while the expression of DE marker *FOXA2* peaked on day 2 and declined slightly on day 7. In addition, significant upregulation of *HNF4A* and *AFP* genes was observed on day 7. These results indicated successful differentiation of hESC toward the hepatoblast lineage.

### VPA Treatment to Promote the Generation of Cholangiocyte-Like Cells

**Figure 2.**
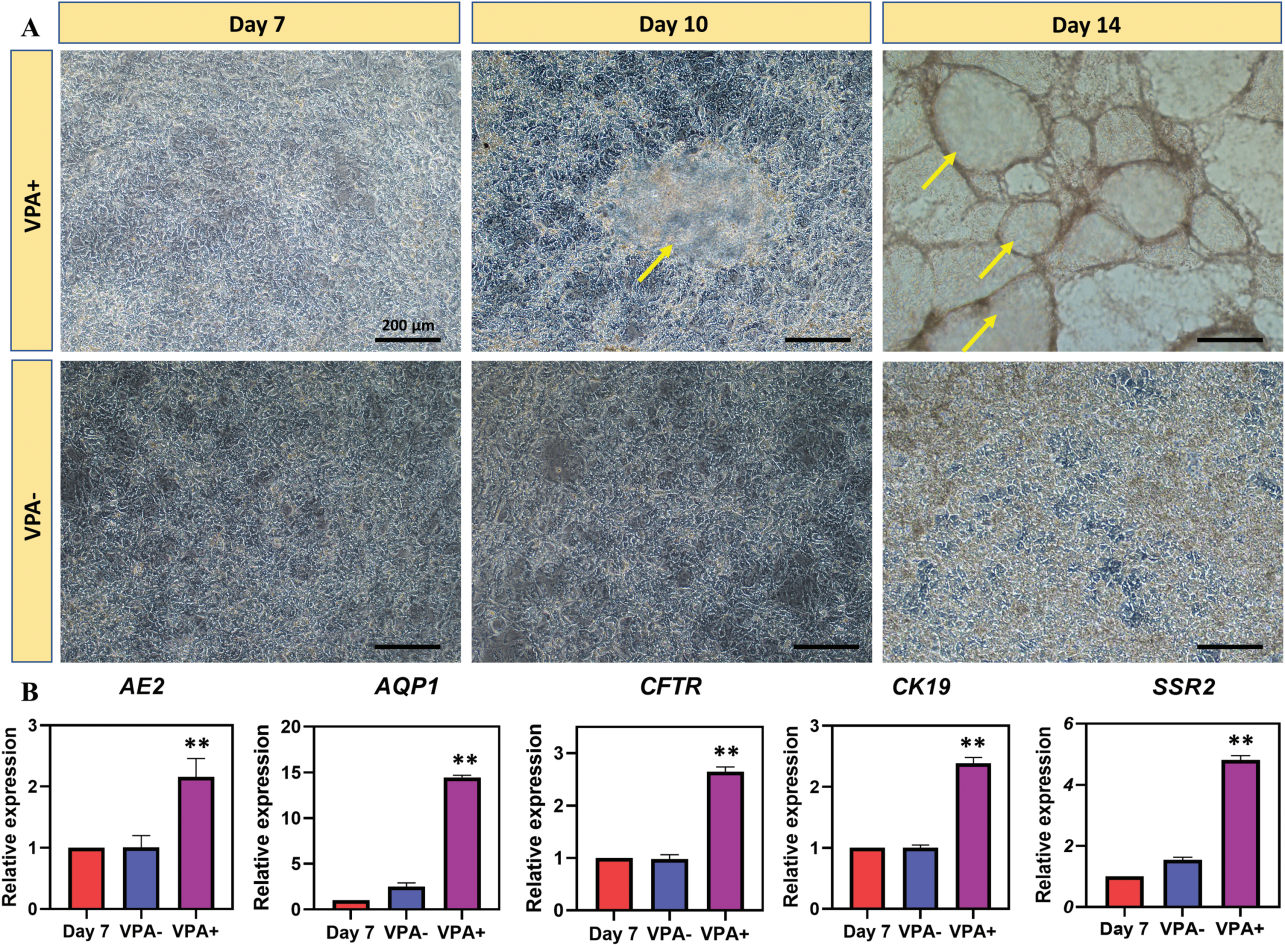
VPA promoted the differentiation of hepatoblasts into cholangiocyte-like cells. (**A**) Phenotypic changes of hepatoblasts with or without VPA treatment. Arrow: cholangiocyte-like cells. (**B**) Real-time PCR showing the expression of cholangiocyte marker genes with or without VPA treatment. (*n* = 3, *P < .05, **P < .01 vs. day 7)

We then assessed whether VPA could be used to drive differentiation of hepatoblasts toward the cholangiocyte lineage. The effects of VPA treatment were comprehensively analyzed in terms of cell morphology, gene and protein expression. To begin with, dramatic morphological changes were observed between VPA- and VPA + groups. In VPA + group, differentiated cells gradually fused and organized to form cystic structure on day 3 and grew into 3D cystic and tubular structure (Fig. 2A), which resembles stem cell-derived biliary cells reported previously.^29^ In contrast, the cells in VPA-group appeared tightly aggregated and resembled hepatocytes.^30,31^ The qPCR results demonstrated significant upregulation of mature biliary markers in VPA + group on day 7, including *AE2*, *AQP1* (a water channel), *CFTR* (a bile acid transporter), *CK19* (a biliary cytokeratin), and *SSR2* (a somatostatin receptor), compared with VPA- and day 7 (hepatoblast) groups (Fig. 2B).

**Figure 3.**
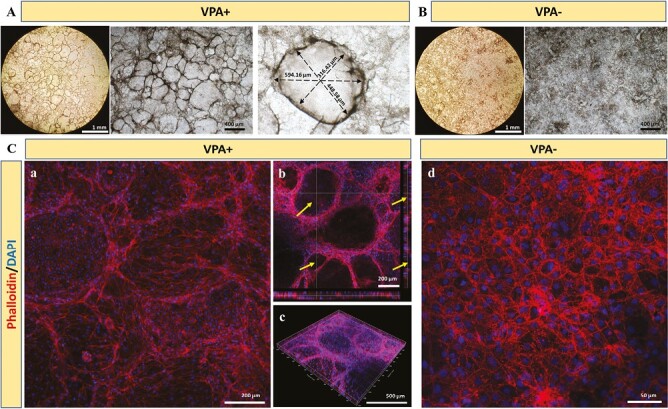
The morphological characterization of the differentiated cells with or without VPA treatment. (**A**) Bright field images of the morphology of cholangiocyte-like cells after the treatment of VPA for 7 days. (**B**) Bright field images of the morphology of cells without VPA treatment. (**C**) Phalloidin and DAPI staining of the differentiated cells with or without VPA treatment. Arrow: biliary cystic-like structures.

**Figure 4.**
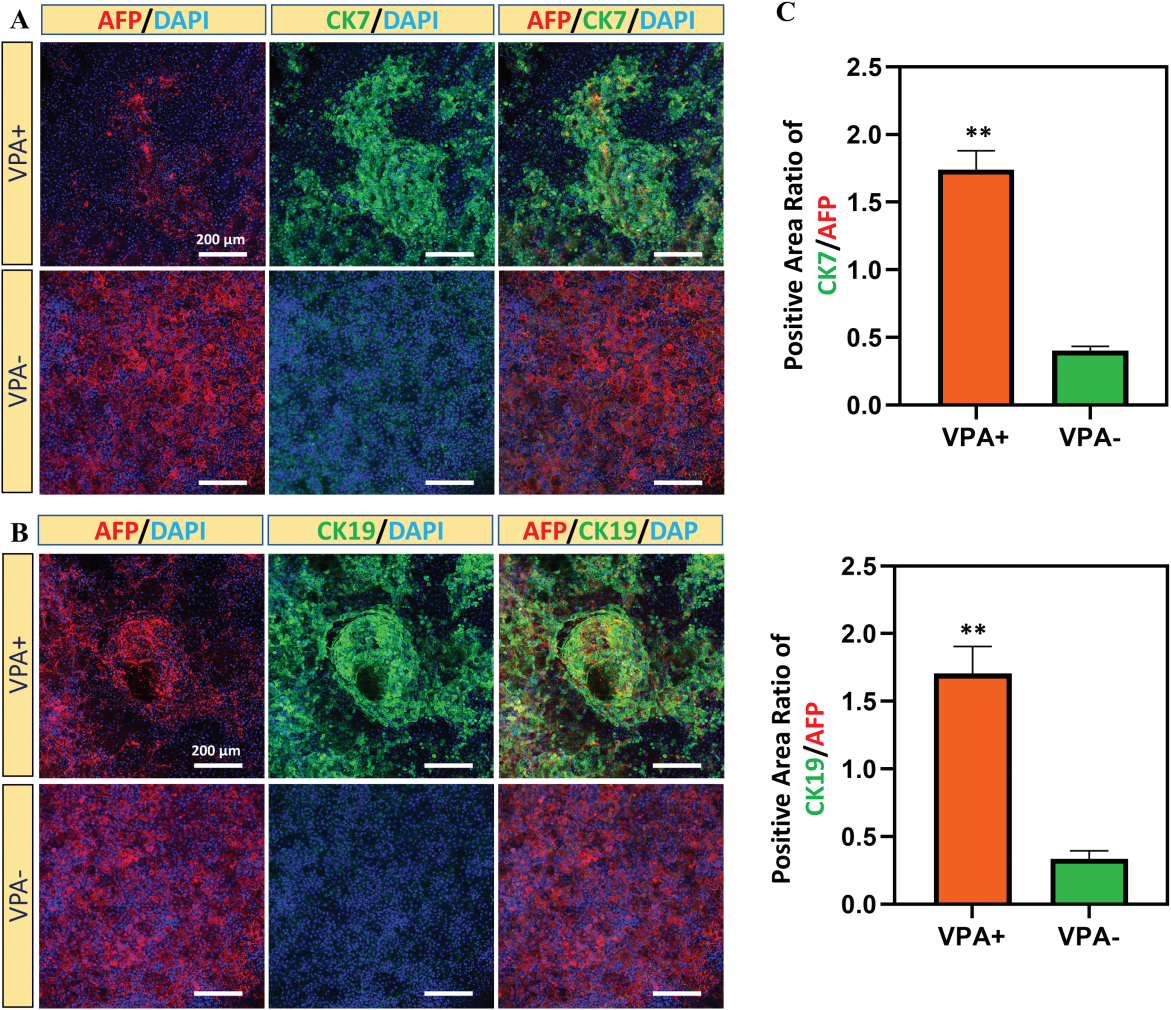
Immunostaining of cholangiocyte marker proteins in differentiated cells with or without VPA treatment. (**A**) Immunostaining of CK7 in differentiated cells with or without VPA treatment. (**B**) Immunostaining of CK19 in differentiated cells with or without VPA treatment. (**C**) Quantification of positive area ratio of CK7, CK19 and AFP in the differentiated cells with or without VPA treatment. (*n* = 3, *P < .05, **P < .01).

In addition, we observed that the number of cystic and tubular structures increased during culture (Supplementary Fig. S1), where the average diameter of cystic structures reached ~531 μm on day 14 (Fig. 3A). Phalloidin immunostaining also confirmed that VPA- (hepatocyte-like) cells and VPA + (cholangiocyte like) cells exhibited different cell morphologies and further validated the formation of 3D cystic and tubular structure in VPA + group (Fig. 3C). To demonstrate the successful differentiation of hepatoblasts to biliary lineage cells under the treatment of VPA, immunostaining of hepatoblast marker AFP and biliary markers CK7 and CK19 was performed (Fig. 4A and B). Reduced AFP expression and increased CK7 and CK19 expression were observed after the treatment of VPA. In particular, the expression of CK7 and CK19 was mainly distributed around the biliary cystic structures. In addition, uptakes of acetylated α-tubulin and rhodamine123 were also observed after VPA treatment (Supplementary Figs. S2 and S3). Taken together, these results demonstrated that VPA promoted the generation of cholangiocyte-like cells from hepatoblasts and the formation of biliary cystic and tubular structures.

### VPA Treatment to Promote Cholangiocyte Differentiation Via Activating Notch Signaling

**Figure 5.**
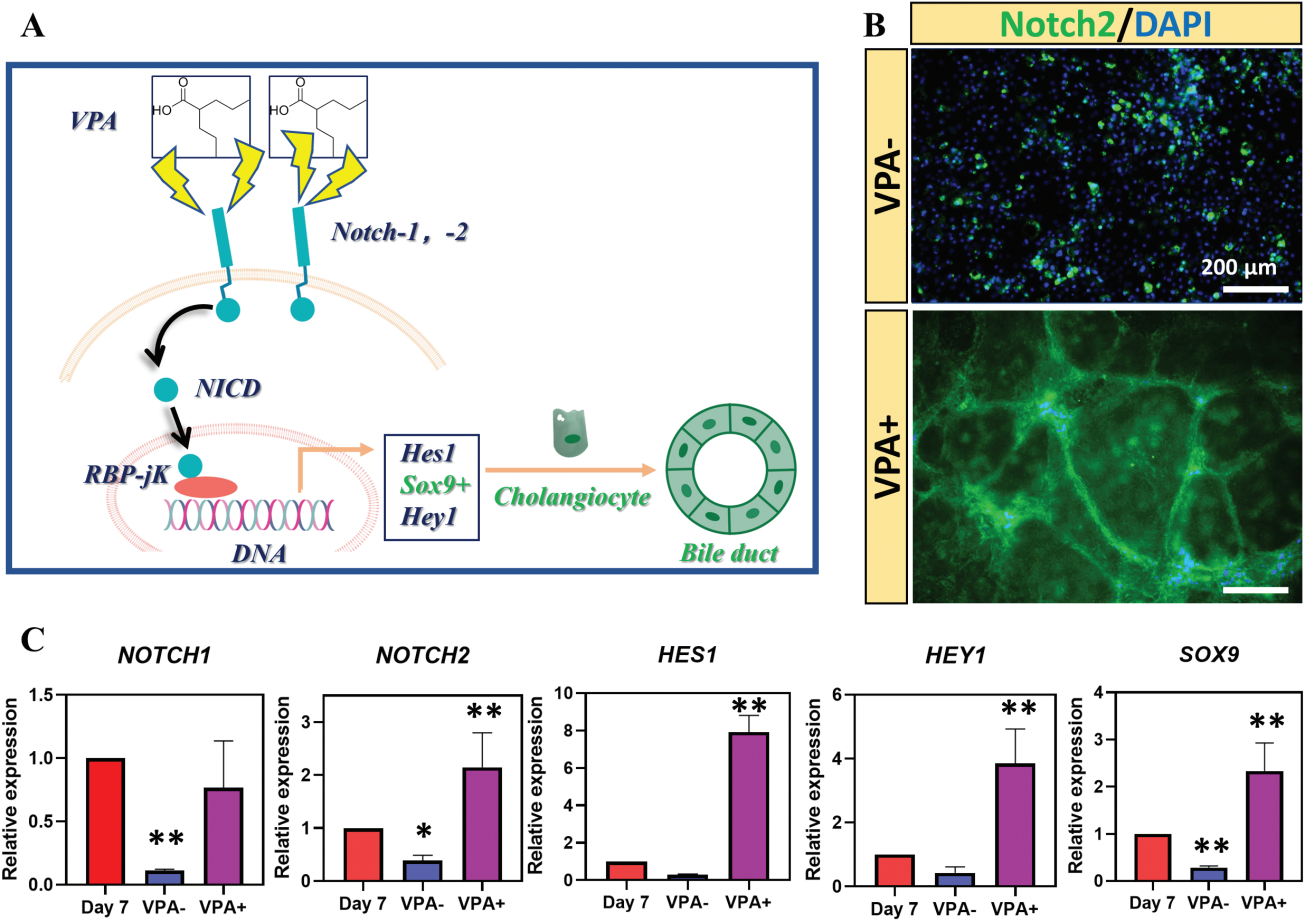
VPA promoted the differentiation of hepatoblasts into cholangiocyte-like cells via activating Notch signaling. (**A**) Schematic diagram of the mechanism of VPA promoting the differentiation of hepatoblasts into cholangiocyte-like cells. (**B**) Immunostaining of Notch2 in differentiated cells with or without VPA treatment. (**C**) Real-time PCR results of the expression of genes in Notch2 signaling pathway. (*n* = 3, *P < .05, **P < .01 vs. day 7).

We further investigated the effect of VPA on promoting hepatoblast differentiation into cholangiocyte-like cells. Previous reports have identified Notch signaling as an important mechanism of biliary lineage commitment.^19,32^ Following the activation of Notch2 receptors, the Notch intracellular domain (NICD) of Notch receptor will translocate to the nucleus, associate with the DNA-binding protein RBPJκ, and mediate changes in gene transcription,^19^ such as Homolog of Hairy/Enhancer-of-Split (HES1) and Hairy/enhancer-of-split related with YRPW motif 1 (HEY1), to promote biliary differentiation (Fig. 5A). After the hepatoblast were treated with VPA, increased expression of *NOTCH2* was observed at both gene and protein levels (Fig. 5B, C). The gene expression level of *NOTCH1* was also significantly higher in VPA + group compared with VPA− group, although the level between that of VPA + group and hepatoblast (day 7) was similar (Fig. 5C). In addition, we also observed increased expression levels of *HES1* and *HEY1*, both of which are known to be the downstream effectors of Notch signaling pathway and regulate biliary lineage differentiation (Fig. 5C). Furthermore, the upregulation of SRY-related HMG box transcription factor 9 (*SOX9*) gene, which was considered a biliary marker and expressed during biliary differentiation,^33^ was observed in VPA + group. This indicated the successful commitment of biliary lineage after the treatment of VPA. Finally, compared with hepatoblast (Day 7), significant downregulation of *NOTCH1*, *NOTCH2*, and *SOX9* genes was observed in the VPA− group. These results suggested VPA promoted biliary differentiation of hepatoblast through activation of the Notch2 signaling pathway and transcriptional activation of downstream HES/HEY family members.

### The In Vivo Construction of Biliary Duct

**Figure 6.**
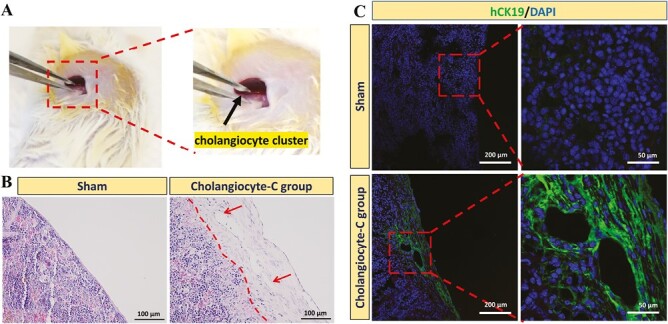
Intrasplenic implantation of hESC-derived cholangiocyte-like cells and analysis after implantation. (**A**) The procedure of introducing cholangiocyte-like cell cluster into the mice spleen. Black arrow: cholangiocyte-like cell cluster. (**B**) HE staining of the spleen section obtained from sham mice and mice receiving implantation of cholangiocyte-like cell cluster (cholangiocyte-c group) 6 weeks after implantation. (**C**) Immunostaining of human-specific CK19 in the spleen section 6 weeks after implantation. The shame-operated mice were used as control.

Next, we tried to evaluate the engraftment of VPA-induced cholangiocyte-like cells in vivo by transplanting them under the splenic capsule of NOD-SCID mice (Fig. 6A). Sham operation was performed as control. At 6 weeks after transplantation, the transplanted cells were found engrafted in the spleen by expressing human specific CK19 (Fig. 6B, C). We also identified biliary duct-like structures that stained positive for hCK19, while hCK19 staining was undetectable in sham-operated spleens, confirming the specificity of the antibodies used.

## Discussion

The limited supply of functional biliary tissues or cells and the failure of animal models to accurately recapitulate human cholangiopathies have been the major obstacles to the development of effective therapies for biliary diseases.^34^ In this study, we explored the use of VPA to activate Notch2 signaling pathway and hence promote hepatoblast differentiation to cholangiocyte-like cells. The differentiated cells expressed biliary lineage-specific protein markers (such as CK7, CK19) and mature biliary gene markers (*AQP1*, *CFTR*, etc.). In addition, differentiated cholangiocyte-like cells were able to form biliary cystic structures and engrafted in vivo.

Previous studies suggested Notch signaling pathway regulated the differentiation of embryonic biliary precursors and promoted the formation of the second biliary layer during development.^19^ Several attempts have been reported to generate cholangiocyte-like cells from hepatic progenitor cells via the activation of Notch signaling pathway.^23,35,36^ However, most of these approaches are time-consuming and involve the use of expensive growth factors/proteins or coculture with a supporting cell type. For example, Ogawa *et al* reported a method to generate cholangiocyte-like cells from hiPSC by coculturing hepatoblasts generated from iPSC with OP9 stromal cells.^37^ This approach required a total culture time of 25 days and administration of growth factors such as TGF-β. Sampaziotis *et al* reported another protocol to generate cholangiocyte-like cells from iPSC-derived hepatoblasts using FGF10, EGF, and Activin.^38^ Compared with previous methods, our small-molecule-based approach could also activate Notch signaling pathway and generate cholangiocyte-like cells, which is more straightforward and less expensive. Besides, our approach requires a relatively short culture time. Treating hepatoblasts with VPA for 7 days resulted in the generation of cholangiocyte-like cells displaying biliary cystic and tubular structures similar to those described in previous studies (Fig. 3).^29^ Moreover, Sampaziotis *et al* reported the diameter of biliary cystic structures formed by isolated primary cholangiocytes were ~547 μm, which is similar to that formed by our hESC derived cholangiocyte-like cells (~531 μm).^39^ Overall, these results suggested VPA treatment is an efficient and relatively inexpensive approach to generate cholangiocyte-like cells. Future studies will focus on optimizing the small-molecule-based differentiation protocols for cholangiocyte production and benchmarking the maturity of the differentiated cells with those generated by growth factor-based approaches.

In this study, the concentration of VPA was chosen according to previous studies about applying VPA to activate Notch2 signaling pathway in neural differentiation.^40,41^ Vukićević *et al* reported that significant upregulation of *NOTCH2* and its downstream effector *HES1* gene was observed in chromospheres cells 24 hours after 2mM VPA treatment. This in turn enhanced the neural differentiation of sympathoadrenal progenitor cells.^27^ VPA was originally approved by the FDA for the treatment of seizures and is now widely applied in neural differentiation. Tao *et al* reported VPA activated Notch signaling pathway via Notch1 in MC3TE‐E1 cells. The downstream effectors *HES1*, *HEY1* were also upregulated after VPA treatment.^42^ While Liu *et al* reported that Notch1 and Notch2 display equivalent function during mice development,^43^ our results showed that only *NOTCH2* upregulation was observed after VPA treatment, while the expression level of *NOTCH1* remained the same (Fig. 5). In contrast, downregulation of both *NOTCH1* and *NOTCH2* was observed in VPA− (hepatocyte differentiation) group. These results demonstrated VPA promoted cholangiocyte differentiation via upregulation of *NOTCH2* instead of *NOTCH1*, while downregulation of both *NOTCH2* and *NOTCH1* occurred during hepatocyte differentiation. Nevertheless, it is of note that one of the limitations of employing a single concentration of VPA in our study is the inability to establish a dose-response relationship.^44^ A dose-response analysis would provide a comprehensive understanding of VPA’s impact on biliary differentiation of hepatoblast, enabling the determination of a minimum effective dose to activate the major signaling pathways (eg, Notch) while mitigating unintended effects on other ones. Consequently, further dosage optimization is necessary to identify the minimum effective dose.

In addition to Notch signaling pathways, expression of marker genes of BMP, Hedgehog, TGF-β, and Wnt signaling pathways was also evaluated by RT-qPCR (Supplementary Fig. S4). The results suggested VPA also promoted the expression of gene (*GLI1*) of the Hedgehog signaling pathway while downregulated *BMP2* expression. This is consistent with previous report by Omenetti et al who showed higher expression of Hedgehog signaling pathway genes in cholangiocyte compared with hepatocyte,^45^ while it is shown that BMP signaling is necessary for liver specification.^46^ No significant difference was observed in *AXIN*, *PTCH1*, and *TGFBR2* gene expression between VPA− and VPA+ groups. These suggested VPA treatment may influence multiple signaling pathways.

Generation of bipotent hepatoblast is prerequisite to the successful promotion of biliary differentiation using VPA. In this study, we have generated hepatoblasts with high expression of AFP gene and protein (Fig. 1C, D). Previous studies have reported the important roles of this state in cholangiocyte differentiation. For example, Lim *et al* reported using *HNF1β* and *FOXA3* to promote mouse fibroblast trans-differentiation into cholangiocyte progenitor cells, and only progenitor cells with high *AFP* expression could differentiate to cholangiocyte-like cells efficiently, while those with low *AFP* expression were associated with low differentiation efficiency.^47^ Therefore, evaluation of *AFP* expression level of hepatoblast may be a key step before cholangiocyte differentiation induced by VPA treatment.

## Conclusion

In this work, we reported the effect of VPA treatment on promoting hepatoblast differentiation into cholangiocyte-like cells for the first time. VPA efficiently activated Notch signaling pathway in hepatoblasts to induce cholangiocyte differentiation. The differentiated cholangiocyte-like cells expressed cholangiocyte marker genes (*AE2*, *AQP1*, *CFTR*, etc.) and proteins (CK19 and CK7), and were able to form biliary cystic structures. Our studies contribute to the generation of cholangiocyte-like cells via a small-molecule-based approach which can facilitate the development of therapies for biliary diseases.

## Supplementary Material

szad079_suppl_Supplementary_Material

## Data Availability

The data underlying this article will be shared on reasonable request to the corresponding author.
