# Supplementary material for: Effect of Valproic Acid on Promoting the Differentiation of Human Embryonic Stem Cells Into Cholangiocyte-Like Cells
Source: Stem Cells Transl Med. 2023 Nov 23;13(2):166–76. doi: 10.1093/stcltm/szad079 (PMC10872666; doi:10.1093/stcltm/szad079)
Supplement: szad079_suppl_Supplementary_Material [file szad079_suppl_supplementary_material.pdf]

Effect of valproic acid on promoting the differentiation of human embryonic stem cells into cholangiocyte-like cells

Supplementary Figures

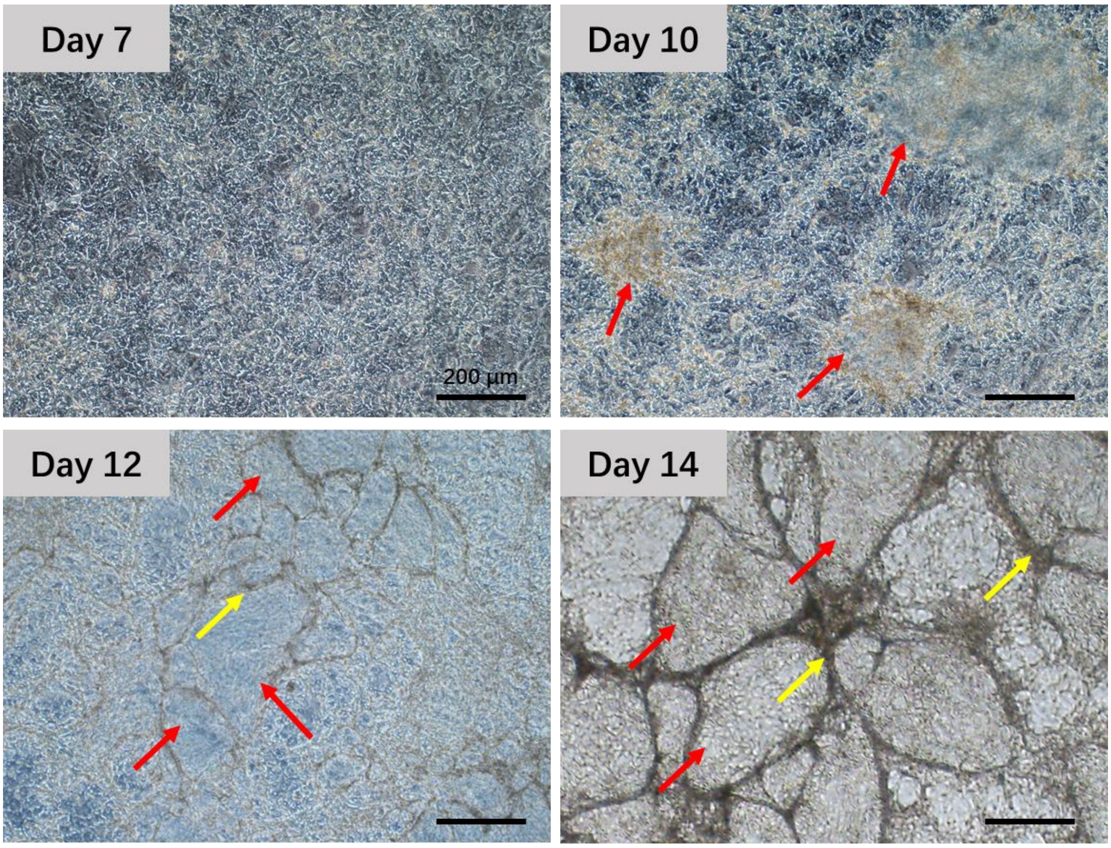

Supplementary Figure 1. Cystic and tubular structures increased during culture. Red arrow: cystic structure. Yellow arrow: tubular structure.

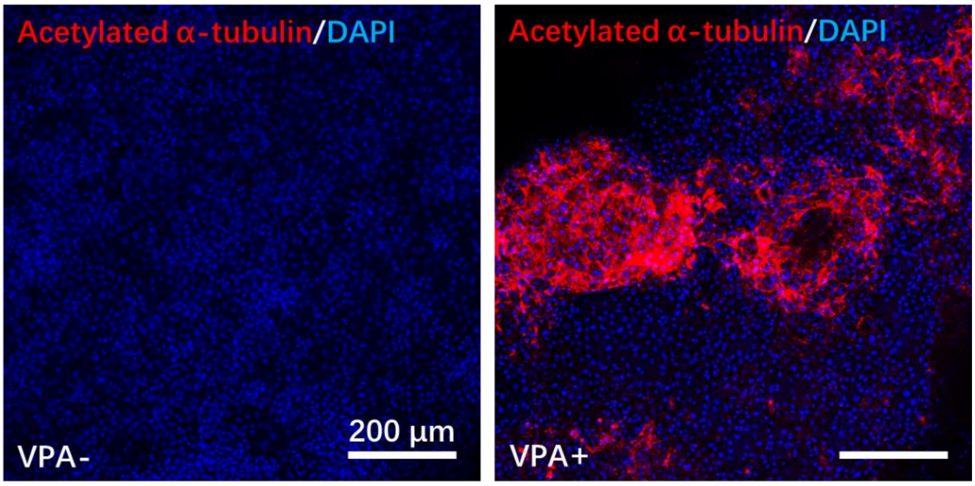

Supplementary Figure 2. Immunostaining of Acetylated α-tubulin in differentiated cells with or without VPA treatment.

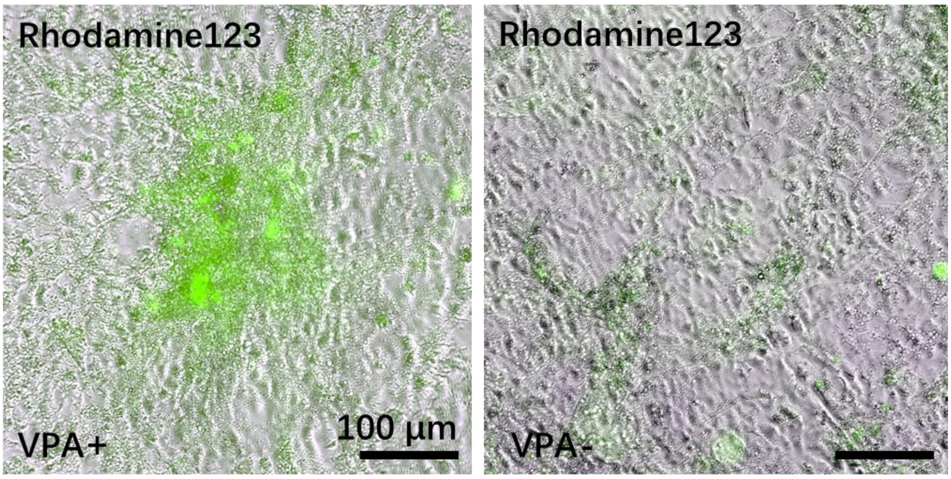

Supplementary Figure 3. The uptake of Rhodamine 123 in differentiated cells with or without VPA treatment.

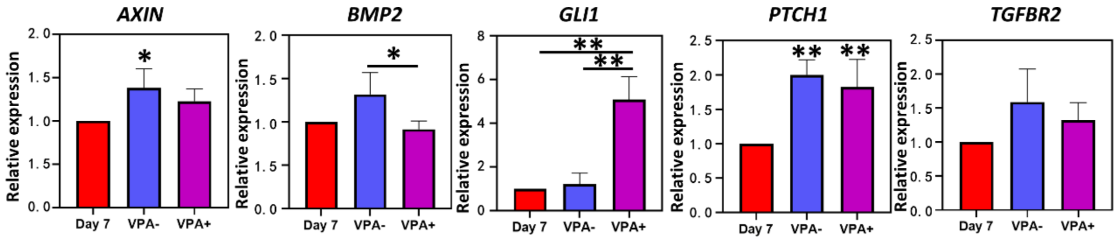

Supplementary Figure 4. Real-time PCR results of the expression of marker genes of BMP, Hedgehog, TGF-β and Wnt signaling pathway. (n=3, * indicates p< 0.05, ** indicates p< 0.01).

Supplementary Tables

Supplementary Table 1. PCR primer used for the quantification of markers

Gene	Forward (5′–3′)	Reverse (5′–3′)
OCT4	CTTGAATCCCGAATGGAAAGGG	GTGTATATCCCAGGGTGATCCTC
FOXA2	GCACTCGGCTTCCAGTATGC	GCGTTCATGTTGCTCACGGA
HNF-4α	GTACTCCTGCAGATTTAGCC	CTGTCCTCATAGCTTGACCT
AFP	AAATGCGTTTCTCGTTGCTT	GCCACAGGCCAATAGTTTGT
GAPDH	ACAACTTTGGTATCGTGGAAGG	GCCATCACGCCACAGTTTC
AE2	TCCTCCCACCACATCCATCA	CTCCTCAATGGTCGGGGTTTC
AQP1	TAACCCTGCTCGGTCCTTTG	AGTCGTAGATGAGTACAGCCAG
CFTR	TGCCCTTCGGCGATGTTTTT	GTTATCCGGGTCATAGGAAGCTA
SSR2	CTTCACCTCGGCAACAATTACT	GGGGAGAATCGCCTGTCAAAC
CK19	GATGCTGTTTGGCATGGGCA	CGTAGATGGGGGTCAGGTCG
NOTCH1	TGGACCAGATTGGGGAGTTC	GCACACTCGTCTGTGTTGAC
NOTCH2	CAACCGCAATGGAGGCTATG	GCGAAGGCACAATCATCAATGTT
HES1	TCAACACGACACCGGATAAAC	GCCGCGAGCTATCTTTCTTCA
HEY1	GAAGTTGCGCGTTATCTGAGC	ATGCGAAACCAGTCGAACTCG
SOX9	AGCGAACGCACATCAAGAC	CTGTAGGCGATCTGTTGGGG

Supplementary Table 2. Antibodies used for the immunostaining

Antibody	Catlog number
OCT4	Abcam: Ab181557
HNF4α	Abcam: Ab41898
AFP	Abcam: Ab253704
CK7	Abclonal: A2574
CK19	Abclonal: A0247
Notch2	Abclonal: A0560
Phalloidin	Abcam: Ab176756
Goat-anti rabbit secondary antibody	Abcam: Ab150077
Goat-anti rat secondary antibody	Abcam: Ab150165
